# Bioremediation of Wastewater Using Yeast Strains: An Assessment of Contaminant Removal Efficiency

**DOI:** 10.3390/ijerph20064795

**Published:** 2023-03-08

**Authors:** Nicoleta-Oana Nicula, Eduard-Marius Lungulescu, Gimi A. Rîmbu, Virgil Marinescu, Viorica Maria Corbu, Ortansa Csutak

**Affiliations:** 1 National R&D Institute for Electrical Engineering ICPE-CA, Splaiul Unirii 313, 030138 Bucharest, Romania; 2 Faculty of Biology, University of Bucharest, Splaiul Independentei 91-95, 050095 Bucharest, Romania; 3 Faculty of Biology, University of Bucharest, 1-3 Aleea Portocalelor, 060101 Bucharest, Romania

**Keywords:** wastewater, yeast, bioremediation, heavy metals, pollutant removal

## Abstract

The main goal of wastewater treatment is to significantly reduce organic compounds, micronutrients (nitrogen and phosphorus) and heavy metals and other contaminants (pathogens, pharmaceuticals and industrial chemicals). In this work, the efficiency of removing different contaminants (COD, NO_3_^−^, NO_2_^−^, NH_4_^+^, PO_4_^3−^, SO_4_^2−^, Pb^2+^, Cd^2+^) from synthetic wastewater was tested using five different yeast strains: *Kluyveromyces marxianus* CMGBP16 (P1), *Saccharomyces cerevisiae* S228C (P2), *Saccharomyces cerevisiae* CM6B70 (P3), *Saccharomyces cerevisiae* CMGB234 (P4) and *Pichia anomala* CMGB88 (P5). The results showed a removal efficiency of up to 70% of COD, 97% of nitrate, 80% of nitrite, 93% of phosphate and 70% of sulfate ions for synthetic wastewater contaminated with Pb^2+^ (43 mg/L) and Cd^2+^ ions (39 mg/L). In contrast, the results showed an increase in ammonium ions, especially in the presence of Pb^2+^ ions. The yeast strains showed a high capacity to reduce Pb^2+^ (up to 96%) and Cd^2+^ (up to 40%) ions compared to the initial concentrations. In presence of a crude biosurfactant, the removal efficiency increased up to 99% for Pb^2+^ and 56% for Cd^2+^ simultaneously with an increase in yeast biomass of up to 11 times. The results, which were obtained in the absence of aeration and in neutral pH conditions, proved a high potential for practical applications in the biotreatment of the wastewater and the recovery of Pb and Cd ions, with a high benefit–cost ratio.

## 1. Introduction

The main goal of wastewater treatment is to remove contaminants and pollutants, so that the water can be safely discharged back into the environment or reused for various purposes. The presence of pollutants (i.e., organic and inorganic compounds, heavy metals) in wastewater can negatively impact the concentration of dissolved oxygen, harm flora and fauna and compromise the quality of water for human consumption and recreational activities. The presence of nitrogen and phosphorus leads to the uncontrolled growth of phytoplankton and toxic and non-toxic algae, causing the phenomenon of eutrophication. The effect of reducing water transparency hinders the penetration of light to underwater vegetation and thus decreases the amount of dissolved oxygen that is produced through photosynthesis [1].

A variety of methods [2,3,4], including physical (e.g., sedimentation, filtration, flotation), chemical (e.g., coagulants, flocculants, disinfectants), physico-chemical (i.e., the adsorption of contaminants by various materials [5,6,7,8]) and biological processes, are commonly used to treat wastewater. Among these, biological methods are considered to be environmentally friendly and have relatively low operating and implementation costs. In the case of biological treatment, microorganisms such as bacteria, fungi, algae, plants and yeasts are used to remove micronutrients [9,10,11], organic compounds [10,12], heavy metals [4,13], etc.

Though yeast species belonging to *Candida* and *Hansenula* genera are known for their potential use in wastewater treatment processes [14], other species, such as *Saccharomyces cerevisiae*, *Kluyveromyces marxianus* or *Pichia anomala,* have been shown to be effective in removing organic compounds or heavy metals from aqueous media or industrial pollutants [15,16,17,18,19]. In general, yeasts are a promising option for wastewater treatment due to several advantages [14,20]. Firstly (i), they can be used in waters with high levels of organic substances (>1000 mg/L) and suspended solids (SS), converting most organic compounds into non-toxic proteins. Yeasts can also reduce the oil content of wastewater by up to 100 times, a feat that cannot be achieved by any other biological treatment method. In a study by Chigusa et al. [21], the treatment efficiency of wastewater from the soybean oil industry was evaluated using nine yeast strains belonging to *Hansenula anomala*, *Candida intermedia*, *Candida schatavii*, *Trichosporon capitatum*, *Candida fluviatilis*, *Candida tropicalis*, *Candida viswanathii*, *Candida pseudolambica* and *Candida hellenica*. The results showed a reduction in the amount of oil in the treated water by up to 96%. Secondly (ii), yeasts have a high capacity for metal accumulation. The removal of heavy metals by yeasts can be achieved through an active (on the surface of viable yeast cells during their growth and development) or passive (on dry yeast) adsorption mechanism [22,23]. Additionally, yeasts are able to reduce the sludge amount and have good settling and dewatering properties (iii). Unlike conventional activated sludge processes, yeast cells form networks of pseudohyphae/hyphae that settle easily and do not require the addition of the coagulation and/or flocculation agents. Most yeast species can grow under semi-anaerobic conditions, requiring less oxygenation (iv). Yeasts used in water treatment require up to 60% less oxygen than activated sludge processes [14]. Finally, the excess of yeast biomass can be used in other biotechnological applications, such as in animal feed, due to its high protein and vitamin content, as well as in the production of biofuels (v) [24].

Biosurfactants are natural surfactants produced by microorganisms such as bacteria, fungi and yeasts, and they offer a biodegradable option for a variety of applications compared to synthetic compounds. They may be used as biocontrol agents in agriculture, the production of cosmetics and pharmaceuticals, food preservation, industrial cleaning and the environmental remediation of pollutants such as heavy metals and organic compounds [25]. Biosurfactants improve the efficiency of wastewater treatment processes by emulsifying and solubilizing the hydrophobic contaminants, reducing the amount of chemicals required for treatment and enhancing the growth rate of microorganisms [26,27,28].

In this study, five yeast strains, including *Kluyveromyces marxianus* CMGBP16 (P1), *Saccharomyces cerevisiae* S228C (P2), *Saccharomyces cerevisiae* CM6B70 (P3), *Saccharomyces cerevisiae* CMGB234 (P4) and *Pichia anomala* CMGB88 (P5), were tested at the laboratory level for their efficiency in reducing or eliminating various contaminants from synthetic municipal wastewater, including COD, NO_3_^−^, NO_2_^−^, NH_4_^+^, PO_4_^3−^, Pb^2+^ and Cd^2+^. We also evaluated the effect of adding a crude biosurfactant obtained from *Yarrowia lipolytica* CMGB32 [29] on yeast biomass yields and the reduction of Pb and Cd ion concentrations in the wastewater. This study was performed in the absence of aeration and at neutral pH levels, which has practical implications for wastewater treatment and heavy metal recovery. Furthermore, the use of a crude biosurfactant to increase removal efficiency is a promising avenue for future research in the field of wastewater treatment.

## 2. Materials and Methods

### 2.1. Growth Curve of Yeast Strains

The yeast strains *Kluyveromyces marxianus* CMGBP16 (P1), *Saccharomyces cerevisiae* S228C (P2), *Saccharomyces cerevisiae* CM6B70 (P3), *Saccharomyces cerevisiae* CMGB234 (P4) and *Pichia anomala* CMGB88 (P5) from the Microorganisms Collection of the Department of Genetics, Faculty of Biology, University of Bucharest were grown in Petri dishes containing solid Yeast Extract–Peptone-Glucose (YPG) medium (consisting of yeast extract 1%, peptone 1%, glucose 2% and agar-agar 2% in distilled water). The strains were incubated at 28 ± 1 °C for 24 h in a CO_2_ Incubator InCuSave MCO 215 LIT (Scientific Instruments, Athens, Greece).

The growth curve of the yeast strains was determined both in liquid YPG medium and in synthetic water contaminated and uncontaminated with Pb^2+^, Cd^2+^ and a mixture of Pb^2+^ and Cd^2+^. This was achieved using 96-well plates containing 400 µL of sample inoculated with 1% of each yeast species, with an initial concentration of 2 McFarland. The experiments were conducted for 24 h at a temperature of 28 ± 1 °C, and optical density values were recorded hour by hour at 570 nm using a Synergy HTX Multimode reader (BioTEK, Winooski, VT, USA). The optical density (OD) measurements at 570 nm were used to determine the degree of yeast development in both uncontaminated and contaminated synthetic water during the experiment.

### 2.2. Preparation of Synthetic Municipal Wastewater

Synthetic wastewater was produced according to the recipe presented in reference [30]: 200 mg/L D-glucose, 200 mg/L Sucrose, 70 mg/L yeast extract, 66.73 mg/L (NH_4_)_2_SO_4_, 10, 91 mg/L NH_4_Cl, 4.43 mg/L KH_2_PO_4_, 21 mg/L MgSO_4_·7H_2_O, 2.68 mg/L MnSO_4_·H_2_O, 30 mg/L NaHCO_3_, 19.74 mg/L CaCl_2_, 0.14 mg/L FeCl_3_·6H_2_O, 455 mg/L NaNO_3_ and 359 mg/L NaNO_2_. For the experiments regarding the removal of heavy metal ions, the synthetic water was contaminated with Pb^2+^ (43 mg/L, (CH_3_COO)_2_Pb) and Cd^2+^ (39 mg/L, Cd(NO_3_)_2_) ions, individually and in a mixture (in the same concentrations as individually) [31]. The heavy metal ion concentrations were precisely determined using ICP-MS analysis.

### 2.3. Determination of Physico-Chemical Parameters of Synthetic Municipal Wastewater

The physico-chemical parameters of the inoculated synthetic water were determined initially and after 24, 48, 96 and 120 h by evaluating the temperature, pH (using a pH meter HI10832, Hanna Instruments, Ins., Woonsocket, RI, USA) and conductivity (using a Multiparameter HI2020-01 Edge, Hanna Instruments, Ins., Woonsocket, RI, USA).

### 2.4. Removal of Pollutants by Yeast Strains

The removal of micropollutants by the selected yeast strains was evaluated using plastic containers with lids, which were filled with 450 mL of uncontaminated and contaminated synthetic water separately. Each sample was inoculated with 1% of each yeast strain, having an initial concentration of 2 McFarland. The samples were incubated at a temperature of 28 ± 1 °C. The integrated time of the experiments was 120 h. In the case of heavy metals, the analyses were carried out at 0, 24 h, 48 h, 96 h and 120 h, and for micropollutants, the analyses were carried out initially and at the end of the experiments.

The capacity to remove organic substances was evaluated by determining the chemical oxygen demand (COD), according to the ISO 6060 standard [32] using Gerhardt Chemical Oxygen Analyzer equipment (C. Gerhardt GmbH & Co., Königswinter, Germany).

The COD removal percentage was determined with the formula:

(1) $$\%\;Removal=\frac{{COD}_{initial}-{COD}_{final}}{{COD}_{initial}}$$

The ability to remove nitrate, nitrite, ammonium, phosphate and sulfate ions was evaluated spectrophotometrically using specific methods and reagents [33], as well as using a Hanna HI83300 Multiparameter Photometer (Hanna Instruments, Ins., Woonsocket, RI, USA).

The removal capacity of Pb^2+^ and Cd^2+^ ions was determined using ELAN DRC-e ICP-MS (Inductively Coupled Plasma Mass Spectrometry) equipment and the calibration curve method for each element separately, for quantitative evaluation.

### 2.5. Determination of the Biomass Amount

The amount of obtained yeast biomass was determined at the end of the experiment by filtering the samples in a vacuum tube through filter paper with pores of 0.45 µm, followed by drying at room temperature.

### 2.6. Determination of the Biosurfactant Influence on the Biomass Production Amount and the Heavy Metal Ion Removal Efficiency

For obtaining the biosurfactant, the yeast *Y. lipolytica* CMGB32 was initially cultivated on YPGA medium for 24 h at 28 °C. From the fresh yeast culture, two colonies were resuspended in sterile distilled water, and the suspension was brought to approximately 1 × 10^8^ cells/mL density. This suspension was used to inoculate 1% Yeast Peptone (YP) broth (10 g/L peptone; 10 g/L yeast extract) supplemented with 1% *n*-hexadecane as a carbon source in a final volume of 450 mL. In order to avoid evaporation of the volatile carbon source, the flask was covered with parafilm and then incubated at 28 °C and 150 rpm for 72 h. At the end of the incubation period, the culture was centrifuged for 10 min at 10,000 rpm and 4 °C, and the recovered supernatant was filtered using sterile Nalgene filter units with 0.2 µm size pores. The cell-free broth obtained after filtering the supernatant was further used as a crude biosurfactant [29,34].

The chemical structure of the obtained biosurfactant was assessed by UV-Vis (UV-Vis Jasco V-570, Jasco Inc., Tokyo, Japan) and FTIR (JASCO FTIR 4200, Jasco Inc., Tokyo, Japan) techniques. The UV-Vis measurements were performed in spectroscopic cells with an optical path of 10 mm on diluted biosurfactant liquid samples, and the FTIR measurements were performed by ATR (attenuated total reflectance) technique on evaporated (at room temperature) biosurfactant samples.

To assess the influence of adding the biosurfactant, a similar experiment as in 2.4 was performed, by introducing a quantity of 5% of the biosurfactant in the recipients.

### 2.7. Statistical Analysis

The analyses were performed in triplicates, and the results were presented as the mean ± standard deviation (SD) of three independent experiments. The statistical significance was analyzed by Student’s *t*-test. A value of *p* less than 0.05 was considered significant. The statistical analysis was performed with Microsoft Excel 2021 software (Microsoft Corporation, Redmond, WA, USA).

## 3. Results and Discussion

### 3.1. Yeast Strains’ Growth Kinetics in Synthetic Medium and in YPG Medium, under Controlled Conditions

**Figure 1:**
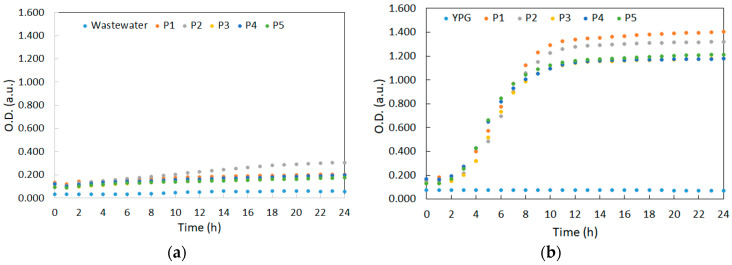
Growth curve of yeast strains in the absence of heavy metals: (**a**) in synthetic wastewater; (**b**) in YPG culture medium.

**Figure 2:**
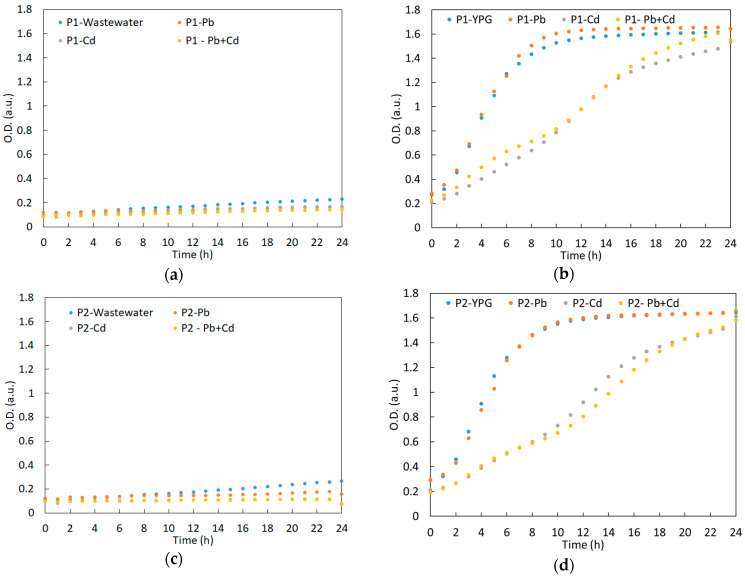

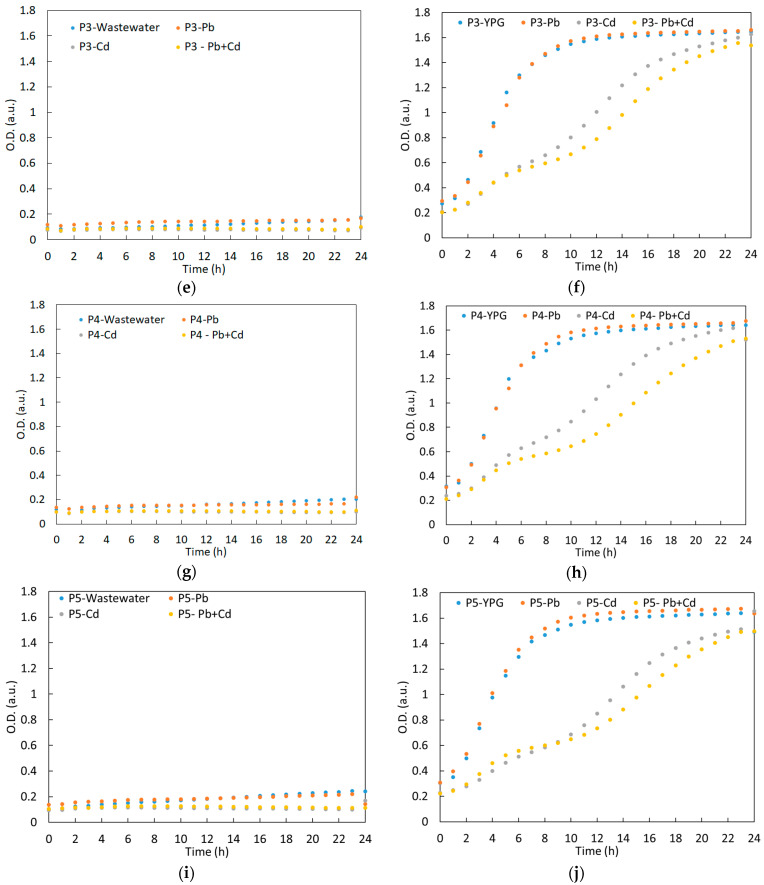
Growth curve of yeast strains in the presence of Pb^2+^, Cd^2+^ and Pb^2+^+ Cd^2+^ in: synthetic wastewater: (**a**) *K. marxianus* CMGBP16 (P1); (**c**) *S. cerevisiae* S228C (P2); (**e**) *S. cerevisiae* CM6B70 (P3); (**g**) *S. cerevisiae* CMGB234 (P4); (**i**) *P. anomala* CMGB88 (P5) and in YPG medium: (**b**) *K. marxianus* CMGBP16 (P1); (**d**) *S. cerevisiae* S228C (P2); (**f**) *S. cerevisiae* CM6B70 (P3); (**h**) *S. cerevisiae* CMGB234 (P4); (**j**) *P. anomala* CMGB88 (P5).

For each yeast strain, growth curves were recorded in both media used during the experiment (synthetic wastewater and YPG), by measuring the optical density at 570 nm over a 24 h period at a temperature of 28 ± 1 °C, both in the absence and presence of heavy metals (Figure 1a,b and Figure 2a,b, respectively).

The composition of both growth media had a significant effect on the timing of the growth phases of the yeast cultures, with higher growth rates observed in the YPG medium compared to the synthetic wastewater (Figure 1). The yeast cultures in synthetic wastewater showed a shorter lag period of 1–2 h compared to 3–4 h in the YPG medium, but the exponential growth phase was slower, occurring over the entire 24 h period with no clear demarcation between the growth and stationary phases. On the other hand, the stationary phase appeared after approximately 10 h in the YPG medium, indicating a rapid growth. This is likely due to the complex composition of nutrients (amino acids, vitamins, organic nitrogen source) in the YPG medium compared to the synthetic wastewater, a fact that highly influences cell growth and multiplication [35].

The strains *K. marxianus* CMGBP16 (P1) and *S. cerevisiae* S228C (P2) showed the best growing rates in both media, with visible differences in the YPG medium, whereas in synthetic wastewater, the differences between the growing curves were less important. The culture growth decreased in the order of *S. cerevisiae* S228C (P2) > *K. marxianus* CMGBP16 (P1) > *S. cerevisiae* CMGB234 (P4) > *S. cerevisiae* CM6B70 (P3) ≥ *P. anomala* CMGB88 (P5) in synthetic wastewater, and *K. marxianus* CMGBP16 (P1) > *S. cerevisiae* S228C (P2) ≥ *S. cerevisiae* CM6B70 (P3), *S. cerevisiae* CMGB234 (P4) and *P. anomala* CMGB88 (P5) in YPG medium, the last three strains following the same growth pattern. Similar profiles of the growth curves were recorded when using media contaminated with Pb^2+^, with the difference that the lag periods were significantly reduced (below 1 h) compared to uncontaminated media (Figure 2). One explanation might reside in the fact that within the first hour of incubation, Pb^2+^ affects cell membrane integrity without an important toxic effect on the cell metabolism, possibly facilitating nutrient uptake from the environment [36].

The controlled contamination of the YPG medium was carried out in order to see the growth behavior of the studied strains under the condition of a high availability of nutrients compared to synthetic wastewater, characterized by a reduced availability of these elements. The growth curves followed the same pattern as in the previous experiment, with higher OD values in the case of the YPG medium compared to the synthetic wastewater (Figure 2a,b). This might be due to the fact that the availability of the heavy metals in the environment plays a crucial role in their toxicity. Since peptone and yeast extract were reported as complexing cadmium [37], it is expected that the Cd^2+^ concentration in the YPG medium would in fact be reduced compared to its concentration in the synthetic wastewater.

However, the growth of the yeast cultures was highly influenced by the heavy metals used for contamination, the strains showing a higher resistance to lead compared to cadmium. Although both metals have a negative effect on the cells, including yeasts, cadmium has a higher level of toxicity, even compared to other heavy metals such as mercury, arsenic or cobalt [38]. Cadmium ions determine cell membrane disruption and the efflux of K^+^ by disturbing the copper and zinc uptake, which impairs the redox balance and mitochondrial respiration and generates free radicals and high levels of lipid peroxidation [39,40]. Moreover, Cd^2+^ also affects vitamin metabolism and causes protein misfolding [41]. Lead mainly affects cell growth by interfering with cell membrane protein synthesis, which determines the leakage of K^+^ and UV_260nm_-absorbing compounds [36,37] and has an inhibitory effect on ammonium uptake [42].

In the case of *S. cerevisiae* strains, some studies have reported the ability to tolerate different concentrations of Cd^2+^ or Pb^2+^. A more recent study investigated the influence of various factors, such as biomass dosage, heavy metal concentration, pH, temperature and the presence of different organic/inorganic nutritional compounds, on the biosorption of lead and cadmium using the fractional factorial design method. The optimum value for the concentration of heavy metals was determined as being 52.5 µg/L. Regarding the impact of the pH value, maximum yields of biosorption could be achieved at pH 5 for Pb^2+^ and pH 6 for Cd^2+^ [43]. For higher concentrations of heavy metals (10–100 mg/mL), the optimal pH values associated with the maximum biosorption of Pb and Cd ions ranged between pH 5.5 and 6.0 [44]. Pasternakiewicz [45] proved that the composition of culture media has an important impact on its ability to grow in the presence of different concentration of cadmium ions. Thus, two *S. cerevisiae* strains were cultivated on brewery wort, respectively, on synthetic yeast-malt culture media enriched with Cd^2+^. According to this study, no significant growth was recorded in the presence of concentrations higher than 200 µM Cd^2+^, probably due to the fact that the adsorption of cadmium is accompanied by the freeing of intracellular potassium, which affects the overall stability of intracellular homeostasis. Regarding the ability of *S. cerevisiae* strains to grow in the presence of Pb^2+^, a study conducted by Chen and Wang [42] proved that at concentrations higher than 5 µM, biomass accumulation was reduced by almost 30% due to the interference of ammonium ion assimilation, although no direct correlation was established between its presence and the cell protein biosynthesis process. Analyzing these data from similar studies, we can affirm that the three *S. cerevisiae* strains used in this study tolerate higher values of Pb^2+^ and Cd^2+^ ions when cultivated both on high-nutrient culture media and under limited nutrient conditions.

Until the present, there have only been a few studies concerning the ability of *Pichia* strains to grow in the presence of heavy metals. Breierova et al. [46] proved that *P. anomala* (formerly known as *Hansenula anomala*, currently renamed *Wickerhamomyces anomalus*) can tolerate small concentrations (less than 1 µM) of Cd^2+^, mainly due to its ability to secrete extracellular glycoproteins with a high content of glutamic acid and mannose, which act as active protective byproducts of cellular metabolism. According to Cai et al. [47], *P. anomala* registered constant growth rates when exposed to Cd^2+^ doses ranging from 10 to 100 mg/L, whereas values of up to 1000 mg/L significantly diminished biomass accumulation. Another study [48] reported six strains of *P. anomala* isolated from polluted lake water with a high ability to accumulate both lead and cadmium ions, among other types of heavy metals. The highest concentration of Pb^2+^ ions tolerated was 200 mM and 100 mM, respectively, for the Cd^2+^ ions. Cultivation in the presence of lead ions was also associated with a significant increase in yeast cell size after 72 h of incubation. However, among *Pichia* species, *Pichia kudriavzevii* has gained more attention regarding its ability to bioaccumulate heavy metals such as cadmium or lead. Members of this species are already known to possess unique metabolic characteristics that ensure its survival in extremely strict cultivation conditions. *P. kudriavzevii* strains can tolerate up to 31 mM Pb^2+^ and 15 mM Cd^2+^ when cultivated on culture media that are poor in organic nutrients, and its resistance is associated with GSH/GSSG ratio variations [49]. These findings correlate with our results, the strain *P. anomala* CMGB88 (P5) showing a good ability to grow in the presence of the tested cadmium and lead concentrations, even under nutrient-restricted conditions in synthetic wastewater.

Another yeast species with a high resistance to stress conditions is *K. marxianus.* The members of this species are thermotolerant and present a versatile metabolism, being able to grow in the presence of a wide range of carbon and nitrogen sources. Although there are still a lot of unknowns concerning the mechanism of tolerance to heavy metals, high biomass accumulation was reported in the presence of 50 mg/mL Pb^2+^ (up to 16 g/L dry biomass) and Cd^2+^ (up to 4 g/L dry biomass), respectively [33]. Moreover, *K. marxianus* cells immobilized in alginate beads were successfully used for removing Pb (II) (up to 62.5 mg/L) from industrial effluents, the highest efficiency being recorded in the presence of neutral pH values and at 35 °C [50].

In the case of the YPG medium and in the presence of cadmium, all yeast strains appear to have a 2-step exponential growth, suggesting a diauxic behavior [51]. In the case of synthetic wastewater, this behavior is visible only for the *K. marxianus* CMGBP16 (P1) strain. In the other strains, after a short exponential period (approx. 4–6 h), the values of O.D. seem to decrease constantly, most probably due to the impact of the heavy metals on cell metabolism.

The simultaneous contamination of the culture media with Pb^2+^ and Cd^2+^ did not seem to have a significant impact on yeast cultures compared to Cd^2+^ contamination, especially on the synthetic water medium. However, the strains *S. cerevisiae* CM6B70 (P3) and *S. cerevisiae* CMGB234 (P4) were most sensitive to the double contamination in the YPG medium, most probably due to the metabolic characteristics related to their origin habitats, i.e., fermented products. Zinc and copper availability plays a major role in fermentative processes. Therefore, the presence of cadmium in the growing media is expected to impair normal metabolic activities.

### 3.2. Growth Curve of the Yeast Strains in Synthetic Wastewater during the Experiment

**Figure 3:**
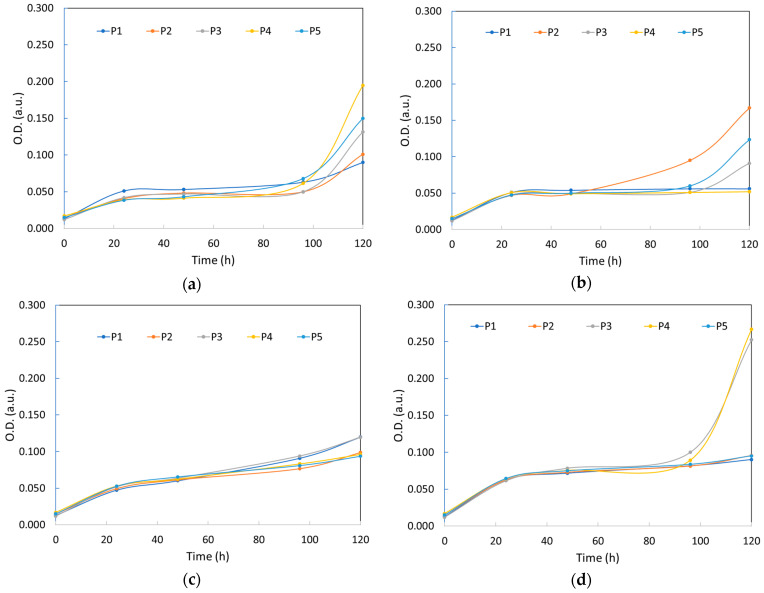
Growth curve of yeast strains under experimental conditions: (**a**) in synthetic wastewater; (**b**) synthetic wastewater contaminated with Pb^2+^; (**c**) synthetic wastewater contaminated with Cd^2+^ ions; (**d**) synthetic wastewater contaminated with Pb^2+^ and Cd^2+^.

Figure 3 shows the growth curves of the yeast strains obtained by optical density measurements performed on synthetic wastewater that was uncontaminated and contaminated with heavy metal ions during the entire experiment (120 h).

The yeast strains grew continuously throughout the experiment, with differences in the growth behavior being a function of the type of metal ions, the lowest values being recorded for the cultures grown in presence of Cd^2+^ (Figure 3c). For all yeast strains, a slow increase in the O.D. was observed during the accommodation period of 96 h, followed by a faster increase up to 120 h. Moreover, the strains *S. cerevisiae* CM6B70 (P3) and *S. cerevisiae* CMGB234 (P4) showed the highest growth rate during the last part of the experiment (Figure 3a–d). Preliminary studies regarding the accumulation of biomass under stress conditions revealed the fact that both *S. cerevisiae* CM6B70 (P3) and *S. cerevisiae* CMGB234 (P4) strains register higher growth rates in the presence of different stress factors (i.e., osmotic stress, extreme pH variations and high temperatures of incubation) [52]. In these circumstances, the accelerated increase in the rate of cell division of the two *S. cerevisiae* strains during the last stages of cultivation in the presence of Pb^2+^ and Cd^2+^ can be explained by the activation of specific stress response mechanisms, which are associated with the expression of the genes encoding the proteins involved in the specific cellular response to the presence of stress factors.

However, these data allowed for the observation of the general trend of yeast development. In order to avoid standard deviation compared to the average values caused by the inhomogeneous distribution of the yeasts throughout the volume, we further performed a quantitative determination of the yeast biomass (Section 3.4).

### 3.3. Determination of the Physico-Chemical Parameters of the Inoculated Synthetic Wastewater during the Experiment

The variations in the conductivity and pH values of the synthetic wastewater inoculated with the five yeast strains were determined over the 120 h of the experiment (Table 1). The temperature was kept constant during the experiment at around 28 ± 0.7 °C. The conductivity values showed a slight tendency to increase with the duration of the experiment and can be correlated with the cell growth stages of the yeast strains used, the highest differences being recorded for *S. cerevisiae* fermentative strains (*S. cerevisiae* CM6B70 (P3) and *S. cerevisiae* CMGB234 (P4)) in the case of lead and lead and cadmium double contaminations, respectively. The higher values of the conductivity observed at the end of the experiment might be an indication of the increase in the number of dead cells and the change of the ion flow (i.e., the release of ions by breaking the structure of cell membranes) [53].

Concerning the pH values, these were quasi-constant, generally between 7 and 8. However, a slight decrease in pH values was observed in the first 48 h of the experiment for the all strains studied, especially in synthetic wastewater and in wastewater contaminated with Pb^2+^ ions, with lower pH values being recorded in the case of strains *K. marxianus* CMGBP16 (P1) (6.28 in the presence of Pb^2+^) and *S. cerevisiae* CM6B70 (P3) (6.41 in synthetic wastewater). This behavior correlates very well with the data from the growth curves and is due to the fact that yeast strains consume nutrients and release organic acids into the environment. Later, after consuming these nutrients, the yeasts use these organic acids as a food source, which leads to an increase in pH values, as also observed in the present experiment [54].

### 3.4. COD Removal and the Amount of Yeast Biomass

**Figure 4:**
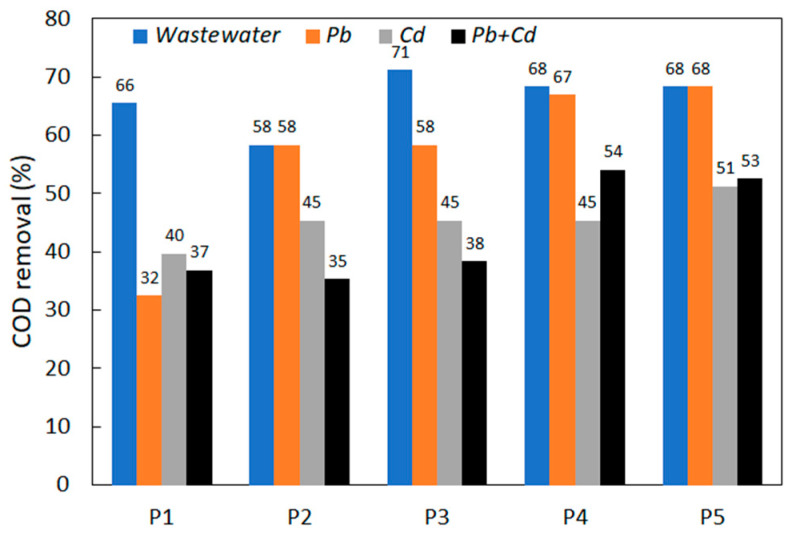
COD removal at the end of the experiment (after 120 h).

The ability of yeast strains to reduce the amount of organic matter was determined by analyzing the variation in chemical oxygen demand (COD) in synthetic wastewater and in synthetic wastewater contaminated with heavy metals (Figure 4). The COD values were determined at the beginning of the experiment and at the end of the experiment (after 120 h).

The comparative analysis of the data showed a general tendency to increase the capacity to reduce COD with the duration of the experiment, up to a maximum of 70% for almost all strains. These results are comparable to those obtained by other authors [55] who studied the COD removal capacity of some yeast species isolated from pharmaceutical wastewater, obtaining a reduction of up to 74% after 72 h of the experiment. Among the species studied by the authors, *P. anomala* showed an efficiency of about 64% in the removal of COD. In our study, the strain *P. anomala* CMGB88 (P5) showed a similar removal efficiency (68%), but at up to 120 h of the experiment. In the same study [55], the authors reported a removal of COD of 27% for *K. marxianus* and 33% for *S. cerevisiae*. According to our study, the *K. marxianus* CMGBP16 (P1), *S. cerevisiae* S228C (P2), *S. cerevisiae* CM6B70 (P3) and *S. cerevisiae* CMGB234 (P4) strains showed a removal efficiency of 66%, 58%, 71% and 68%, respectively, but at up to 120 h of the experiment.

In the case of heavy metal contamination, the removal of COD was generally lower than that observed in the case of uncontaminated wastewater. It can also be observed that the reduction capacity was higher in the case of wastewater contaminated with Pb^2+^ than in the case of wastewater containing Cd^2+^. These results are well correlated with our previous results on the growth curves and the larger amount of biomass formed in the presence of lead compared to cadmium (Table 2), confirming the augmented toxicity of Cd^2+^ ions on the yeast cells. Thus, in the case of synthetic wastewater contaminated with Cd^2+^ ions (both individual and mixed with Pb^2+^), the amount of biomass formed was about 50–60% lower than in the case of uncontaminated and Pb^2+^-contaminated synthetic wastewater.

### 3.5. Nutrient Removal in Inoculated Synthetic Wastewater

**Figure 5:**
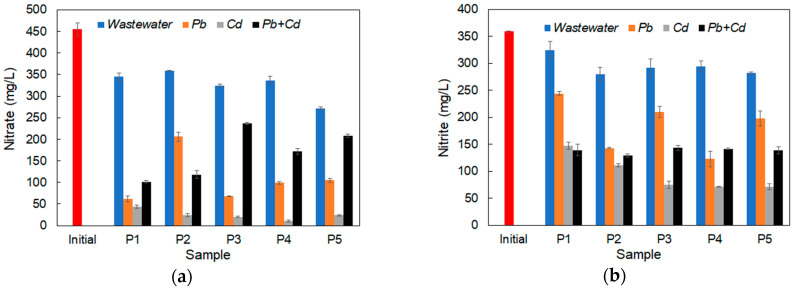

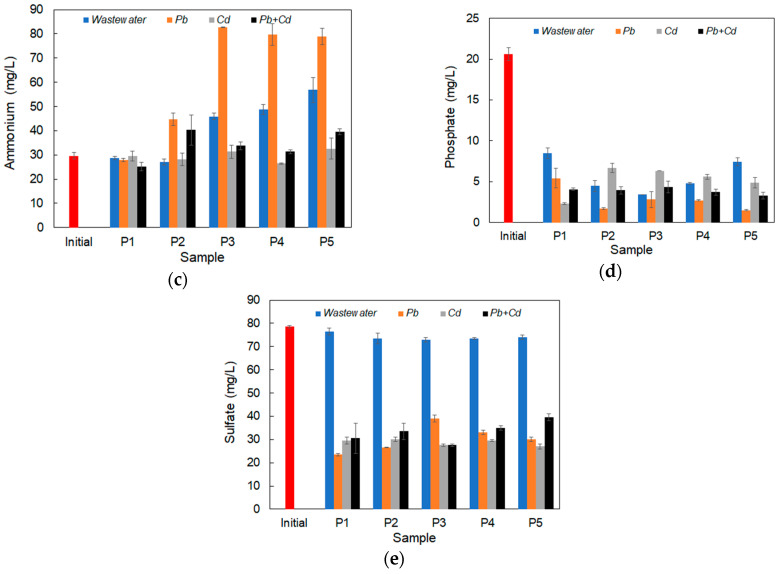
Removal efficiency of nutrients from synthetic wastewater after 120 h: (**a**) nitrate ions; (**b**) nitrite ions; (**c**) ammonium ions; (**d**) phosphate ions; (**e**) sulfate ions.

Nutrient pollution consists of an over-enrichment of water with nitrogen and phosphorus, producing eutrophication of aquatic ecosystems with adverse effects such as the accumulation of organic carbon, reduced light penetration and the loss of submerged aquatic vegetation. It also causes an imbalance in nutrient proportions, creating favorable conditions for the development of toxic algae and reducing the amount of oxygen in the water [56]. Figure 5 shows the variation in nitrate, nitrite, ammonium, phosphate and sulfate ions in the inoculated synthetic wastewater after 120 h of the experiment. In general, a significant decrease in the concentration of these ions was observed compared to the initial value, with higher values in the samples contaminated with Cd^2+^ ions. This fact suggests a higher uptake of nitrogen substrates required for cells in order to overcome the metabolic stress induced by the presence of cadmium in the environment.

In the case of nitrate ions (Figure 5a), an efficiency of their removal of between 20–40% can be observed in wastewater without the presence of heavy metals, the greatest reduction being observed in the case of the *P. anomala* CMGB88 (P5) strain and the lowest in the case of the *S. cerevisiae* S228C (P2) strain. In the case of synthetic wastewater contaminated with metal ions, the removal behavior of nitrate ions is dependent on the type of metal ions used. The highest removal rate was observed in the case of wastewater contaminated only with Cd^2+^ ions, up to 97% (with an average value, on all strains, of 94 ± 2.33 mg/L), followed by the wastewater contaminated with Pb^2+^ ions, up to 85–86% (*K. marxianus* CMGBP16 (P1) and *S. cerevisiae* CM6B70 (P3)), and that contaminated with both types of ions, up to 74–77% (*K. marxianus* CMGBP16 (P1) and *S. cerevisiae* CMGB234 (P4)), respectively.

A similar trend was also observed in the case of nitrite ions (Figure 5b), although the removal efficiency was lower. Thus, in the case of synthetic wastewater, the reduction rate ranged from 9% for the *K. marxianus* CMGBP16 (P1) strain to 22% for the *S. cerevisiae* S228C (P2) strain. These values were much lower than those obtained in the case of wastewater contaminated with: Cd^2+^ ions (between 60%—*K. marxianus* CMGBP16 (P1) and 80%—*S. cerevisiae* CMGB234 (P4) and *P. anomala* CMGB88 (P5)), Pb^2+^ ions (between 32%—*K. marxianus* CMGBP16 (P1) and 65%—*S. cerevisiae* CMGB234 (P4)) and that contaminated with both ion species (60–64%).

In the case of ammonium ions (Figure 5c), the behavior of all yeast strains was different from that observed for the other types of nutrients. Thus, the removal rate was insignificant. Moreover, an increase in the concentration of ammonium ions was observed, especially in the case of Pb^2+^ contamination for the strains *S. cerevisiae* S228C (P2), *S. cerevisiae* CM6B70 (P3) and *S. cerevisiae* CMGB234 (P4) and *P. anomala* CMGB88 (P5), most probably due to one of the main effects of lead toxicity—a reduction in the ammonium uptake from the environment [42]. A less important augmentation of ammonium ions was also observed in noncontaminated synthetic wastewater for the strains *S. cerevisiae* CM6B70 (P3), *S. cerevisiae* CMGB234 (P4) and *P. anomala* CMGB88 (P5). This might be related to the composition of the medium. According to Zikanova et al. [57], the formation of ammonia by yeast cells represents a mechanism related to impaired mitochondrial oxidative catabolism and the activation of peroxisomal oxidation, as a strategy of survival under starvation conditions. In the case of the wastewater inoculated with the *K. marxianus* CMGBP16 (P1) strain, the determined amount of ammonium ions was similar to the one recorded in the initial synthetic water, suggesting a high ability of this strain to adsorb lead ions.

The higher concentrations of ammonium ions observed in the case of synthetic wastewater without heavy metals and that contaminated with lead correlate very well with the amount of biomass formed (Table 2) and, therefore, with the increase in yeast biomass per unit volume. Since the concentration of ammonium ions was determined at the end of the experiment (after 120 h), it is possible to initially observe a decrease in the concentration of ammonium ions (which represents an important source of assimilable nitrogen for yeast development) [58].

The capacity to reduce phosphate ions (Figure 5d) was also influenced by the experimental conditions used (yeast strains, type of heavy metal contaminants). In general, in the presence of both Pb^2+^ and Cd^2+^ ions, the amount of removed phosphate ions was higher than in the case of synthetic wastewater without these metals. Thus, in the case of uncontaminated wastewater, there was a decrease in the concentration of phosphate of between 59% for *K. marxianus* CMGBP16 (P1) and 83% for *S. cerevisiae* CM6B70 (P3); in the presence of Pb^2+^, between 73% for *K. marxianus* CMGBP16 (P1) and 93% for *S. cerevisiae* CM6B70 (P3) and *P. anomala* CMGB88 (P5); in the presence of Cd^2+^, between 67% for *S. cerevisiae* CM6B70 (P3) and 88% for *K. marxianus* CMGBP16 (P1); and in the presence of Pb^2+^ + Cd^2+^, between 79% for *S. cerevisiae* CM6B70 (P3) and 83% for *P. anomala* CMGB88 (P5). The highest reduction rates obtained in the case of lead contamination correlate with the metabolic stress induced by its presence in the environment, leading to an increased accumulation of phosphate into the yeast cell [59]. On the other hand, cadmium causes more damage to the general cell metabolism, affecting the uptake of various compounds, including phosphate, from the culture media. These results are of particular importance for decreasing the effect caused by water eutrophication, since the studied yeast strains showed the important ability to simultaneously reduce nitrogen and phosphorus compounds. The outcome is emphasized by the fact that, at present, the specialized literature presents only a few yeast species that are capable of removing phosphate ions from water [60,61,62].

The high rates of sulfate ions’ reduction (Figure 5e) in the wastewater contaminated with Pb^2+^ and Cd^2+^ (up to 30-fold) compared to uncontaminated wastewater should also be noted. Thus, the most important results were obtained in the case of the wastewater contaminated with Pb^2+^ ions, between 50% for *S. cerevisiae* CM6B70 (P3) and 70% for *K. marxianus* CMGBP16 (P1), followed by the wastewater contaminated with Cd^2+^, between 61% *S. cerevisiae* S228C (P2) and 65% for *K. marxianus* CMGBP16 (P1), and the wastewater contaminated with both types of ions, between 49% for *P. anomala* CMGB88 (P5) and 64% for *S. cerevisiae* CM6B70 (P3). In the case of metal-free synthetic wastewater, the removal efficiency of sulfate ions was between 3% for *K. marxianus* CMGBP16 (P1) and 7% for *S. cerevisiae* CM6B70 (P3). The removal of sulfate ions may be due to the enhanced requirement of sulfates for the synthesis of sulfur-containing amino acids, methionine, cysteine and their derivatives [63], important compounds in protein synthesis.

Overall, although the nutrient removal efficiency varied depending on the yeast strain and the type of added heavy metal, the strain *K. marxianus* CMGBP16 (P1) showed an increased removal efficiency of nitrate, nitrite, phosphate and sulfate ions in the presence of Pb^2+^ and Cd^2+^ (added individually or as a mixture). Similar results were obtained in the case of *S. cerevisiae* CMGB234 (P4) and *S. cerevisiae* CM6B70 (P3) strains, with the note that in their case, the best results for all nutrients except for ammonium ions were obtained mainly in the presence of Pb^2+^ ions. The strain *P. anomala* CMGB88 (P5) showed the important ability to remove phosphate and sulphate ions, especially in the presence of the Pb^2+^ and Cd^2+^ mixture.

In conclusion, correlating the data with previous results on biomass accumulation, COD and nutrient removal, we can conclude that *K. marxianus* CMGBP16 (P1), *S. cerevisiae* CMGB234 (P4) and *P. anomala* CMGB88 (P5) strains have the highest potential for removing Pb^2+^ and Cd^2+^ ions, both as independent pollutants as well as in a mixture, thus representing a viable solution for the decontamination of wastewater and the prevention of eutrophication.

### 3.6. Removal of Heavy Metal from Inoculated Wastewater

Environmental pollution with heavy metals is becoming more and more of a problem and has become a great concern due to the adverse effects it causes worldwide. These inorganic pollutants are released into the water, soil and atmosphere through agricultural and industrial activities, through the improper disposal of waste, fertilizers and pesticides [64]. Heavy metals are natural elements that have a density greater than 5 g/cm^3^. They are toxic and non-biodegradable and therefore pose a serious threat to the environment and most organisms, including humans, through bioaccumulation [65]. Heavy metals such as Cd, Cr (VI), Pb, As, Hg, Cu, Ni, Zn, etc. interfere with various physiological pathways within the body, including that of humans, and thus cause many diseases. Cases have been observed with Cd, which causes obstructive pulmonary disease, kidney damage and cardiovascular disease. Cr (VI) causes lipid and protein degradation, mutations and cancer. Hg causes pulmonary edema, chemical colitis, kidney and CNS damage, and Pb causes scoliosis and lordosis, insomnia, neural damage and so on [65].

For environmental protection, certain limits have been established for heavy metals in water, above which they are considered to be pollutants, namely: Pb^2+^—0.1 mg/L and Cd^2+^—0–0.005 mg/L [65].

There are a variety of mechanisms for the removal of heavy metals from aqueous solutions by microorganisms and higher plants. The cellular response to the presence of metals includes various processes, such as biosorption through cellular biomass, active cell transport, binding to cytosolic molecules, sequestration in cellular capsules, precipitation and redox reactions, as well as protein–DNA adduct formation and protein induction of stress [66].

Table 3 shows the removal capacity of Pb and Cd ions (individually and in a mixture) of the yeast strains used in the experiment. The amount of heavy metals was determined by ICP-MS measurements at different time periods (0, 24, 48, 96 and 120 h) based on calibration curves.

Of the two types of heavy metals used for contamination (Pb, Cd), the largest amount of metal removed was in the case of contamination with Pb ions, with up to 96% for the *K. marxianus* CMGBP16 (P1) strain, and in the case of the double contamination of Pb and Cd, of up to 95% for the *S. cerevisiae* CMGB234 (P4) strain. After the first 24 h of the experiment, the quantified removal capacity was between 50–69% for synthetic wastewater contaminated only with Pb ions and between 63–95% for the water contaminated with both types of ions, respectively. After 120 h of the experiment, the most efficient strains in the removal of Pb ions (individual) were *K. marxianus* CMGBP16 (P1) with 96%; followed by *S. cerevisiae* CMGB234 (P4) and *P. anomala* CMGB88 (P5), both with 90%; *S. cerevisiae* CM6B70 (P3) with 89%; and *S. cerevisiae* S228C (P2) with 86%. In the case of wastewater contaminated with both types of ions, the removal efficiency decreased from S. cerevisiae CMGB234 (P4) and P. anomala CMGB88 (P5), both with 95%, to *S. cerevisiae* CM6B70 (P3) with 94%, *K. marxianus* CMGBP16 (P1) with 91% and *S. cerevisiae* S228C (P2) with 84%.

In the case of wastewater contaminated with Cd^2+^, the removal efficiency was significantly lower than that of Pb^2+^ ions, confirming the results obtained from the growth curve and the amount of biomass formed, as well as the higher toxicity of cadmium against the studied yeast strains. However, it was obvious that the removal of Cd ions ranged between 15–40% (after 120 h) in the case of wastewater contaminated only with Cd^2+^ and between 32–39% for the mixture with Pb^2+^, respectively. Among the five studied strains, the most effective were *P. anomala* CMGB88 (P5) with 26%, followed by *S. cerevisiae* CM6B70 (P3) with 24%, *S. cerevisiae* CMGB234 (P4) with 22% and *K. marxianus* CMGBP16 (P1) and *S. cerevisiae* S228C (P2), both with 10%.

The high efficiency of Pb^2+^ ions’ removal could be due to the secretion of exopolysaccharides of extracellular polymeric substances (EPS) by yeast cells. EPS contain a variety of organic components that in turn have a highly branched chemical structure and functional groups such as hydroxyl and carboxyl groups [67]. This spatial structure and complex composition allow for the adsorption and chelation of Pb^2+^ ions, but also of Cd^2+^, reducing their toxicity [68]. The ability to remove Pb^2+^ is also most likely enhanced by the presence of phosphate ions, which are considered effective elements for removing lead from water [68].

Moreover, all the strains used during the study showed a high potential for removing Pb^2+^ ions of up to 96% compared to the initial concentration, much higher than the values obtained in other studies, indicating an approx. 70–80% removal rate using the *S. cerevisiae* strains [69]. The strains *K. marxianus* CMGBP16 (P1), *S. cerevisiae* CMGB234 (P4) and *P. anomala* CMGB88 (P5) can be considered the best candidates for cadmium and lead removal, combined with biomass accumulation.

Our results indicated that the removal capacity of cadmium and lead is influenced by the type of strain used in wastewater treatment, by the type of heavy metal used to contaminate the water and also by the duration of the experiment.

The efficiency of the process could be improved by increasing the concentration of yeast inoculum in the synthetic wastewater, as shown in similar works, in which an increment from 5 × 10^8^ CFU/mL to 22 × 10^8^ CFU/mL resulted in an enhancement of the removal efficiency of approx. 60% [69]. Another alternative would be using natural adjuvants such as biosurfactants, which are able to form complexes with the metal ions or to adsorb them, reducing their toxicity in the environment [70].

### 3.7. The Influence of Y. lipolytica Biosurfactant on the Reduction Capacity of Organic Compounds and Heavy Metals

Based on the experimental results presented above, the strains *K. marxianus* CMGBP16 (P1), *S. cerevisiae* CMGB234 (P4) and *P. anomala* CMGB88 (P5) were used to study the effect of the biosurfactant obtained from *Y. lipolytica* CMGB32 on the amount of biomass formed, COD and the efficiency of Pb^2+^ and Cd^2+^ removal. The experiments were carried out under similar conditions as the previous ones, with the evaluation of the efficiency of heavy metal removal being recorded after 24, 48, 96 and 120 h, and with the amount of biomass formed determined at the end of the experiment, after 120 h. We also considered only the lead and cadmium ions’ separate contamination, since their cumulated effect on yeast metabolism did not present any surprising results.

Because the obtainment procedure and characterization of the biosurfactant from *Y. lipolytica* was presented in detail in reference [29], in this paper, we tried to generate some additional information about the ionic character and chemical structure of the biosurfactant.

The tests concerning the ionic character of the biosurfactant (not shown here), using the double diffusion technique in agar [71,72] in comparison with sodium dodecyl sulfate (SDS, anionic) and BaCl_2_ (cationic), revealed that the biosurfactant has a rather nonionic character or is neutral.

**Figure 6:**
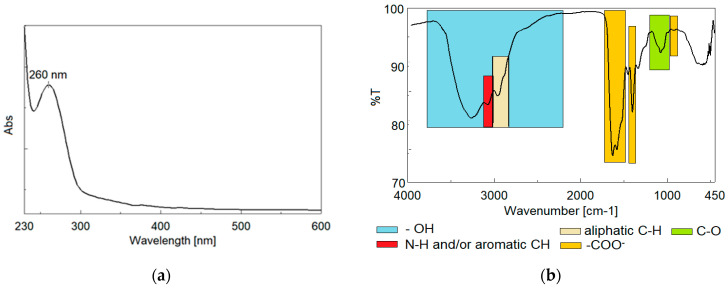
Spectroscopy analysis of biosurfactant: (**a**) UV-Vis; (**b**) FTIR.

The UV–Visible spectrum (Figure 6a) of the biosurfactant showed the presence of an absorption peak at about 260 nm, most probably indicating the presence of aromatic amino acids in its structure [73]. The absorption peak is similar to those obtained by other studies for biosurfactants [74].

The FTIR spectrum (Figure 6b) recorded for the biosurfactant showed vibration bands characteristic of some aromatic amino acid compounds, confirming the UV-Vis data. Thus, the FTIR spectrum is characterized by a broad band between 2280–3700 cm^−1^ due to the vibration of OH bonded from COOH [75]. This band also overlaps with characteristic vibrations of both N-H and aromatic bonds (3070 cm^−1^) from amino acids [76] and aliphatic C-H bonds (2960 cm^−1^) [77]. The multipeak band between 1480–1766 cm^−1^ contains characteristic peaks of the carboxyl groups COO^−^ (1581, 1575 cm^−1^) [75], but also of the aromatic ring (1518—1540 cm^−1^). Peaks characteristic of the COOH vibration from acids can also be observed at 1400 cm^−1^ and 927 cm^−1^. The amide-type vibration from the amino acid is observed at 1331 cm^−1^, and the broad peak at 992–1175 is specific to the vibration of the C-O bond [75].

The chemical structure of *Y. lipolytica* biosurfactants seems to be strongly influenced by the nature of the carbon source used as a substrate [78,79,80,81]. These literature data correlate very well with our results, indicating that the biosurfactant produced by *Y. lipolytica* CMGB32 is essentially a lipoprotein, and the protein part has a large number of aromatic amino acids in its composition.

The addition of the *Y. lipolyitca* biosurfactant into synthetic wastewater inoculated with the three yeast strains had a beneficial effect on the removal efficiency of Pb and Cd ions (Table 4), especially in the case of wastewater contaminated with Cd^2+^ ions, for which an increased removal of up to 56% for the strain *K. marxianus* CMGBP16 (P1) was observed compared to only 15% in the case of synthetic wastewater without a biosurfactant. Removals of up to 49% and 51% were also obtained for the *P. anomala* CMGB88 (P5) and *S. cerevisiae* CMGB234 (P4) strains, significantly higher than in the case of wastewater without a biosurfactant (39% and 30%, respectively). In the case of wastewater contaminated with Pb ions, the addition of a biosurfactant also leads to the increase in their removal efficiency of up to 97%, 99% and 98%, compared to 96%, 90% and 90% (in the case of wastewater without a biosurfactant) for the *K. marxianus* CMGBP16 (P1), *S. cerevisiae* CMGB234 (P4) and *P. anomala* CMGB88 (P5) strains, respectively.

**Figure 7:**
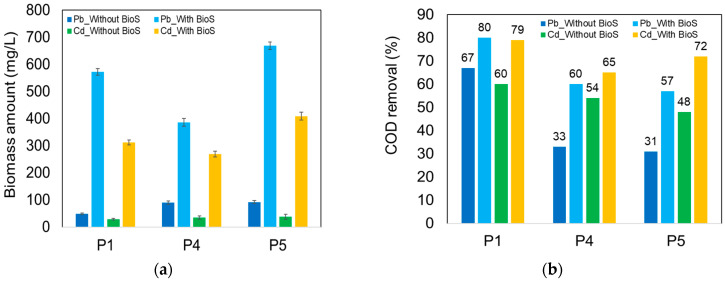
The influence of biosurfactant on (**a**) the amount of yeast biomass; (**b**) removal of COD.

The increase in removal efficiency can be correlated with the significant increase in the amount of yeast biomass in the presence of a biosurfactant (Figure 7a), indicating a better assimilation of the nutritive compounds from the environment, due to a reduction in the metal ions’ toxicity as a result of their complexation by the biosurfactant. The carboxyl groups from the structure of the biosurfactant interact with Pb^2+^ and Cd^2+^ ions from wastewater, leading to their chelation and the formation of metal–biosurfactant complexes [82,83]. Under these conditions, a higher increase in biomass (up to 11 times) was observed in the case of the *K. marxianus* CMGBP16 (P1) strain, both in the presence of Pb^2+^ and Cd^2+^ ions, whereas for P5, we recorded an increment of approx. 7 times in the presence of Pb^2+^ and of 10 times in the presence of Cd^2+^, respectively.

The larger amount of biomass in the presence of the biosurfactant also provides an explanation for the increase in COD removal efficiency from wastewater. Thus, in the case of wastewater contaminated with Pb^2+^ ions, we determined an increase in COD removal efficiency compared to wastewater without a biosurfactant, of up to 82% for *S. cerevisiae* CMGB234 (P4), 79% for *P. anomala* CMGB88 (P5) and 19% for *K. marxianus* CMGBP16 (P1). In the case of the presence of Cd^2+^ ions, the increase in COD removal efficiency compared to wastewater without a biosurfactant was of up to 50% for *P. anomala* CMGB88 (P5), 32% for *K. marxianus* CMGBP16 (P1) and 20% for *S. cerevisiae* CMGB234 (P4).

## 4. Conclusions

The efficiency of removing different contaminants from synthetic municipal wastewater was tested using five different yeast strains: *Kluyveromyces marxianus* CMGBP16 (P1), *Saccharomyces cerevisiae* S228C (P2), *Saccharomyces cerevisiae* CM6B70 (P3), *Saccharomyces cerevisiae* CMGB234 (P4) and *Pichia anomala* CMGB88 (P5).

The contaminant removal efficiency (of COD, NO_3_^−^, NO_2_^−^, NH_4_^+^, PO_4_^3−^, SO_4_^2−^, Pb^2+^ and Cd^2+^) was dependent on the inoculated yeast strain and the type of heavy metal used for water contamination, and it correlated very well with the amount of yeast biomass formed. Experimental results showed a removal of up to 70% of COD and 97% for nitrate, 80% nitrite, 93% phosphate and 70% sulfate ions from wastewater contaminated with Pb^2+^ (43 mg/L) and Cd^2+^ ions (39 mg/L). Ammonium ions were found to be resistant to removal, with some strains even showing an increase in concentration, especially due to Pb and Cd toxicity effects.

The studied yeast strains also showed a high capacity to reduce Pb^2+^ (up to 96%) and Cd^2+^ (up to 40%) ions, compared to the initial concentration. The use of a biosurfactant produced by *Y. lipolytica* CMGB32 further increased the removal efficiency of Pb (up to 99%) and Cd (up to 56%) ions as well as that of COD (up to 80%), these processes being closely correlated with a significant increase in the amount of biomass (up to 11 times).

The experimental conditions used in the study, such as the absence of aeration and neutral pH values, suggest that the biotreatment of wastewater using yeast strains is a practical and cost-effective approach.

Overall, the results of this study provide a positive future perspective for the use of yeast strains in the biotreatment of wastewater and the recovery of Pb and Cd ions, particularly in industries that generate large volumes of wastewater. The study may also have implications for the growing body of research on innovative, sustainable and eco-friendly methods for treating wastewater and reducing environmental pollution.

## Figures and Tables

**Table 1:** Variations in the physico-chemical parameters of inoculated synthetic wastewater.

Time (h)	Synthetic Wastewater					
	Parameters	P1	P2	P3	P4	P5
0	Conductivity (µS·cm^−1^)	1767	1767	1746	1767	1754
	pH	7.74	7.77	7.74	7.73	7.74
24	Conductivity (µS·cm^−1^)	1750	1754	1744	1751	1751
	pH	6.34	6.54	6.52	6.53	6.53
48	Conductivity (µS·cm^−1^)	1797	1745	1756	1762	1758
	pH	6.44	6.45	6.41	6.44	6.45
96	Conductivity (µS·cm^−1^)	1797	1773	1775	1784	1790
	pH	7.00	6.42	6.84	7.18	7.14
120	Conductivity (µS·cm^−1^)	1798	1722	1777	1784	1789
	pH	7.47	6.52	7.2	7.35	7.46
	Synthetic wastewater + Pb^2+^					
0	Conductivity (µS·cm^−1^)	1770	1776	1766	1767	1768
	pH	7.53	7.51	7.5	7.5	7.49
24	Conductivity (µS·cm^−1^)	1754	1763	1761	1753	1762
	pH	6.28	6.52	6.54	6.43	6.49
48	Conductivity (µS·cm^−1^)	1765	1762	1771	1767	1774
	pH	6.35	6.47	6.47	6.47	6.52
96	Conductivity (µS·cm^−1^)	1776	1762	1792	1798	1799
	pH	6.42	6.71	6.22	7.02	6.64
120	Conductivity (µS·cm^−1^)	1781	1778	1801	1810	1788
	pH	6.46	7.19	6.35	7.36	6.84
	Synthetic wastewater + Cd^2+^					
0	Conductivity (µS·cm^−1^)	1780	1776	1781	1778	1770
	pH	7.7	7.71	7.70	7.71	7.72
24	Conductivity (µS·cm^−1^)	1774	1780	1781	1782	1767
	pH	7.43	7.43	7.44	7.45	7.48
48	Conductivity (µS·cm^−1^)	1774	1782	1781	1772	1759
	pH	7.48	7.43	7.45	7.50	7.53
96	Conductivity (µS·cm^−1^)	1773	1790	1785	1743	1747
	pH	7.64	7.68	7.70	7.68	7.72
120	Conductivity (µS·cm^−1^)	1781	1756	1760	1772	1743
	pH	7.65	7.67	7.62	7.63	7.69
	Synthetic wastewater + Pb^2+^ + Cd^2+^					
0	Conductivity (µS·cm^−1^)	1704	1775	1724	1749	1747
	pH	7.91	7.67	7.56	7.49	7.47
24	Conductivity (µS·cm^−1^)	1781	1777	1774	1776	1770
	pH	7.33	7.39	7.35	7.34	7.34
48	Conductivity (µS·cm^−1^)	1782	1779	1787	1793	1785
	pH	7.58	7.56	7.45	7.41	7.47
96	Conductivity (µS·cm^−1^)	1797	1777	1816	1826	1806
	pH	7.71	7.67	7.55	7.59	7.49
120	Conductivity (µS·cm^−1^)	1772	1784	1802	1742	1730
	pH	7.59	7.50	7.46	7.51	7.44

**Table 2:** The amount of yeast biomass at the end of the experiment.

Yeast Strain	Biomass Amount (mg/L)			
	Synthetic Wastewater	Synthetic Wastewater/Pb^2+^	Synthetic Wastewater/Cd^2+^	Synthetic Wastewater/Pb^2+^ + Cd^2+^
P1	61.00 ± 5.20	49.25 ± 1.30	28.38 ± 0.55	32.25 ± 0.70
P2	80.38 ± 5.25	88.25 ± 1.40	29.75 ± 0.70	34.38 ± 0.85
P3	96.75 ± 0.80	71.63 ± 7.85	30.13 ± 0.25	34.50 ± 1.50
P4	91.00 ± 2.60	90.00 ± 2.00	35.13 ± 0.05	34.50 ± 0.70
P5	97.88 ± 2.85	92.38 ± 3.75	37.88 ± 1.15	39.63 ± 0.30

**Table 3:** Efficiency of heavy metals’ removal.

Time (h)	Removal Efficiency (%)																			
	P1				P2				P3				P4				P5			
	Pb	Cd	Pb/Cd		Pb	Cd	Pb/Cd		Pb	Cd	Pb/Cd		Pb	Cd	Pb/Cd		Pb	Cd	Pb/Cd	
			Pb	Cd			Pb	Cd			Pb	Cd			Pb	Cd			Pb	Cd
24	70	10	65	23	51	2	70	27	45	2	81	26	66	0	85	26	68	27	84	3
48	78	10	81	30	63	3	74	32	58	12	85	32	68	3	87	32	75	32	85	11
96	92	10	82	30	85	10	83	33	81	24	85	32	88	23	87	37	85	34	89	26
120	96	15	92	39	86	20	85	33	89	26	94	33	90	30	95	40	90	39	95	32

**Table 4:** Pb and Cd ions’ removal efficiency with biosurfactant.

Time (h)	Removal Efficiency (%)					
	P1		P4		P5	
	Pb	Cd	Pb	Cd	Pb	Cd
24	72	14	70	12	69	16
48	82	26	79	22	84	26
96	93	43	94	40	88	37
120	97	56	99	51	98	49

## Data Availability

The data presented in this study are available on request from the corresponding author.
